# Targeting the cholinergic and endocannabinoid systems as a therapeutic intervention for core and associated phenotypes in the autism model; a systematic review

**DOI:** 10.47626/2237-6089-2024-0791

**Published:** 2025-09-18

**Authors:** Princewill Sopuluchukwu Udodi, Godson Emeka Anyanwu, Roseline Ebube Udodi, Damian Nnabuihe Ezejindu

**Affiliations:** 1 Department of Anatomy College of Medicine University of Nigeria Nigeria Department of Anatomy, College of Medicine, University of Nigeria, Enugu Campus, Nigeria.; 2 Department of Anatomy College of Health Sciences Nnamdi Azikiwe University Nigeria Department of Anatomy, College of Health Sciences, Nnamdi Azikiwe University, Nnewi Campus, Nigeria.; 3 Department of Anatomy Faculty of Biomedical Sciences Kampala International University Uganda Department of Anatomy, Faculty of Biomedical Sciences, Kampala International University, Uganda.

**Keywords:** Endocannabinoid, cholinergic, agonist, antagonist, autism

## Abstract

**Objective:**

Autism spectrum disorder (ASD) is a neurodevelopmental disorder that has been linked to dysregulation in the cholinergic and endocannabinoid (EC) systems. This study systematically reviews the present literature on treatment strategies aimed at enhancing the activity of both systems in ASD models.

**Methods:**

We performed a systematic evaluation of literatures that investigated the effects of different therapeutic interventions on the components of the cholinergic and EC systems in ASD models, following the guidelines provided by the Preferred Reporting Items for Systematic Reviews and Meta-Analyses (PRISMA) checklist. Four databases were searched: Google Scholar, Web of science, EMBASE, and MEDLINE/PubMed, for articles published from August 2012 to February 2023. References cited in the selected research papers were also examined. Twelve papers (five on the cholinergic system, six on the EC system, and one on both) were reviewed in this study of prior work on relevant treatment strategies that impact these systems. The paper cites a total of 77 studies.

**Results:**

The majority of research revealed that different therapeutic interventions downregulated cannabinoid 1 (CB1) receptors, and the system’s hydrolyzing enzymes and upregulated EC, alpha 7 nicotinic acetylcholine receptor (α7-nAChR), and ACh signaling molecules. Regulation of the components of the cholinergic and EC systems by these therapies generally enhanced behaviors in ASD models.

**Conclusion:**

It is possible that the therapeutic interventions assessed in one or both of these systems may be effective for treating the core ASD-associated phenotype. The benefits of the therapeutic interventions reviewed in this study merit further investigation in randomized, blinded, placebo-controlled clinical trials.

## Introduction

The diagnosis of autism spectrum disorder (ASD), a neurodevelopmental disorder that affects social communication and interaction throughout life, is marked by limited and/or repetitive interests and/or behaviors that first appear before the age of three.^1^ ASD now includes a number of disorders that were grouped together under the category of pervasive developmental disorders (PDDs) in the first generation of medical classifications. However, because it is a spectrum condition, there is also a high degree of heterogeneity in its phenotypic manifestations, which are linked to a wide range of intellectual and language development levels, intra-individual differences in cognitive profiles, and a history of comorbidity with other developmental disorders and psychiatric conditions.^2,3^ According to Rogala et al.^4^ and Yasuda et al.,^1^ autism is a diverse condition with a complex etiology involving many different elements, including genetic, epigenetic, environmental, and immunological components.

The cholinergic and endocannabinoid (EC) systems’ neurological signals constitute the body’s vast regulatory network, which keeps physiology and homeostasis in check. ASD has been linked to dysregulated EC and cholinergic systems.^5-7^ Many physiological processes and neuroadaptive reactions depend critically on the cholinergic system and the EC system. These are involved in numerous stages of brain development and encompasses nociception, reward, learning and memory, movement control, and endocrine function.^8,9^ Acetylcholine (ACh) and ECs influence synaptic transmission and plasticity in the central nervous system (CNS) by modulating neurotransmission. By activating type 1 cannabinoid receptors (CB1Rs), which are predominantly found at presynaptic locations, and nicotinic acetylcholine receptors (nAChRs), these neurotransmitters specifically control the release of both excitatory and inhibitory neurotransmitters.^10,11^ The idea of a bidirectional crosstalk between the nicotinic cholinergic and EC systems has gained support over time from a growing body of preclinical research. In multiple brain areas, nAChRs and CB1Rs exhibit close overlap and are widely expressed in the CNS.^12,13^ When nicotine and delta-9-tetrahydrocannabinol (Δ9-THC), the main psychoactive components of tobacco and cannabis, respectively, are given to animals, they cause a number of common pharmacological effects, including hypothermia, induction of anti-nociception, rewarding effects, dependence, and impairment of locomotion.^14^

One common neuromodulatory system is the EC system. This system has a significant impact on development of the CNS, synaptic plasticity, and the body’s reaction to internal and external stressors.^15^ The EC system is made up of endogenous cannabinoids (ECs), cannabinoid receptors, and the enzymes that synthesize and degrade ECs.^15^ Although CB1Rs are the most dominant kind of cannabinoid receptors, some cannabinoids also activate CB2 receptors, transient receptor potential (TRP) channels, and peroxisome proliferator-activated receptors (PPARs). Cannabinoid receptor interactions enable exogenous cannabinoids, such as tetrahydrocannabinol and cannabidiol, to exert their biological effects. The two endogenous cannabinoids that have been investigated the most are 2-arachidonoyl glycerol (2-AG) and arachidonoyl ethanolamide (AEA- anandamide).^15^ Many neurological illnesses are attributed to etiologies involving changes in EC system functionality.^16^ The observation that the EC system is highly involved in regulation of social and emotional reactivity as well as in modulation of behaviors that are frequently altered in ASD, such as learning and memory processes, seizure susceptibility, and circadian rhythm regulation, provides indirect evidence of this system’s involvement in ASD.^17,18^ Different autism models exhibit significant decreases in the levels of AEA and 2-AG,^19^ while valproic acid (VPA)-exposed autistic animals showed abnormal phosphorylation of the CB1Rs in the dorsal striatum, hippocampus, and amygdala.^20^

Given the large density of cholinergic synapses found in the neocortex, limbic system, thalamus, and striatum, it is likely that cholinergic transmission plays a key role in memory, learning, attention, and other higher-order brain functions.^21^ Numerous research directions point to additional cholinergic system functions in the general homeostasis and plasticity of the brain. As a result, current research on cognitive and social deficiencies heavily relies on the brain’s cholinergic system.^21^ The neurotransmitter molecule ACh, acetylcholine receptors (AChRs), choline acetyltransferase (ChAT), and acetylcholinesterase (AChE) are all components of the cholinergic system. These molecules play dual roles in the brain, acting as neurotransmitters and neuromodulators. They are crucial for arousal, motivation, memory, attention, and homeostasis maintenance. In response to neuronal inputs, the majority of innate and adaptive brain cells release or express these molecules on their surfaces. ASD-related core behavioral deficits may result from dysregulation of this neural system communication. A number of preclinical ASD animal models seem to have dysregulated cholinergic systems.^6,7^ Meyza and Blanchard^22^ describe the BTBR mouse model for ASD, which is an inbred mouse strain that has an *Itpr3* gene deletion. Mice with BTBR exhibit abnormal nicotinic cholinergic neurotransmission, repetitive behaviors, and social communication problems.^23^ In BTBR mice, nicotine treatment reduced these distinctive behaviors associated with ASD.^23^ Similar results were observed when donepezil, an AChE inhibitor, was given to BTBR mice in another study.^24^

Targeting the cholinergic and EC system, a number of agonists, antagonists, and inhibitors have been developed to help with the fundamental behavioral deficits associated with ASD. This review will address the various therapeutic interventions for dysregulated cholinergic and EC systems in ASD, offering a comprehensive and current systematic overview of the literature on potential therapies that could improve the activities of the cholinergic and EC systems in ASD. Which molecules influence the defective behaviors in animal models of ASD, and which agonists and antagonists affect the components of the cholinergic and EC systems? Improved methods for regulating these systems in ASD may result from a greater understanding of the varied roles played by pharmacological compounds and other behavioral therapy approaches.

## Methods

We conducted our systematic literature review in December 2023 using the Preferred Reporting Items for Systematic Reviews and Meta-Analyses (PRISMA) approach. This was the guideline for approval of the study by the review and research ethics committee at the Department of Anatomy, College of Medicine, University of Nigeria, Enugu Campus.

The following questions were asked to guide the review: 1) How safe and effective were the treatments, and how well did they enhance the components of the cholinergic and EC systems? 2) What impact did the therapies have on behavioral deficits linked to the systems’ activities in the ASD models? 3) Which key methods were used to assess the improvement in behavior?

Four databases were searched: Google Scholar, Web of Science, EMBASE, and MEDLINE/PubMed. The search strategy for the databases was developed based on terms found in the title or abstract, using descriptors related to the EC and cholinergic systems (the systems’ receptors, signaling molecules, and enzymes) as well as descriptors related to autism (autistic, autism, Asperger, transgenic autism, BTBR mouse model, sodium valproate autism model, and pervasive developmental disorder). Articles in any language were considered in the analysis of eligibility; no language restrictions were imposed during the selection process. The search operators “AND” and “OR” were used, in addition to enclosing descriptors in quotation marks. Terms linked to cholinergic, EC system, ASD, autistic animal model, and autism were clustered together using the “OR” operator. These two groups of linked sentences were then combined using the “AND” operator.

Papers published between August 2012 and February 2023 that satisfied the inclusion requirements were selected. Book chapters, abstracts, studies on animals, and studies on other illnesses or alterations associated with symptoms and indicators similar to those shown in the autism model were all disregarded as irrelevant to the subject. Articles discussing enhancers, agonists, or antagonists of the cholinergic and EC system components of ASD models were considered in this study.

The first screening was done by reading the abstracts and titles of the papers that were found in the database searches. Articles that were deemed appropriate for the proposed topic were then read in full. Following the screening procedure, we examined the papers’ references to determine if any additional relevant research met the eligibility requirements. Three authors independently and concurrently carried out the search and one experienced author vetted the selected articles. The most knowledgeable and experienced author made the final decision about whether or not to include a given study, always making sure to verify the qualifying requirements. A total of 17, 12, eight, six, and five articles were found by searches conducted on the MEDLINE/PubMed, Google Scholar, Web of Science, and EMBASE databases and in the reference lists of the reviewed papers, respectively. These were reduced to 9, 2, and 1 items, respectively, by exclusion of those that did not meet the inclusion criteria. After additional screening, 12 papers were found to entirely match the inclusion criteria.

The method for extracting data from the trials involved completing a standardized information sheet for each. After three reviewers had extracted the scientific information, a fourth reviewer verified the data that had been gathered. Any disagreements were discussed and decided upon by the reviewers and writers.

## Result

**Figure 1:**
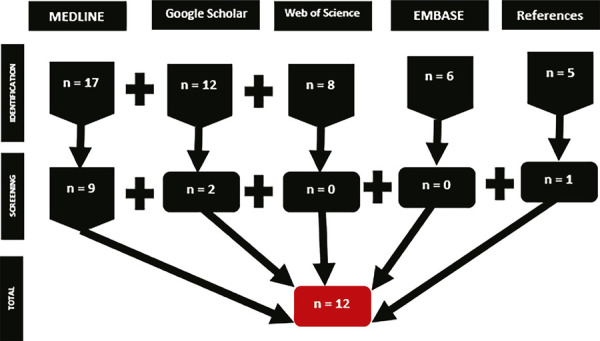
Flowchart according to the Preferred Reporting Items for Systematic Reviews and Meta-Analyses (PRISMA) illustrating selection of studies of targeting the cholinergic and endocannabinoid systems for therapeutic interventions for core autism spectrum disorder (ASD)-associated phenotypes in the ASD model: a systematic review.

The initial search results identified 48 articles. Initial screening disqualified 36 studies for failing to meet the inclusion criteria, either because they did not investigate any components of the cholinergic or EC systems, because they involved primary research on a non-specific ASD animal model, because they were studies looking into the therapeutic potentials of other brain signaling systems, or because they were investigations into the symptoms of other disorders and conditions that share some traits with the ASD animal model. Following this process, three articles from Google Scholar, nine articles from MEDLINE/PubMed, and one article from the references of reviewed papers were selected, while none of the articles from web of science and EMBASE were selected. Twelve papers in all were picked for the final analysis (Figure 1). The systematic review analyzed four, three, two, one, one, and one studies conducted in the United Arab Emirates, United States of America, China, Egypt, Ireland, and Italy, respectively.

This review identified several therapeutic interventions with a positive impact on the components of the EC system and the cholinergic system. E100, also known as H3 receptor antagonist and AChE inhibitor, was administered to VPA-C57BL/6 and BTBR autistic models. E100 downregulated AChE in the hippocampus of VPA-C57BL/6 and BTBR autistic models and upregulated anandamide, with no significant difference in the level of 2-AG when compared to untreated VPA-C57BL/6 and BTBR autistic models.^25,26^ The impact of E100 on the various components of the EC and cholinergic systems reversed the various phenotypes associated with the core ASD symptoms.^25,26^ E100 enhanced sociability and social novelty index, reduced the number of buried marbles, increased time spent in and the number of entries into the open arm, and decreased the percentage escalation of shredded nestlets.^25,26^ Cannabidivarin, JZLI84, and environmental enrichment, which are known to directly or indirectly modulate CB1Rs, were shown to down-regulate CB1Rs with a positive impact on social activities, cognition, and repetitive behaviors.^27-29^ JZLI84 downregulated CB2 receptors in the hippocampus and prefrontal cortex with decreased escape latency and time of platform crosses, increased sociability and novel preference index, decreased number of buried marbles, and decreased grooming time.^28^ Acetaminophen, URB59, and PF3845 are known as either direct or indirect modulators of CB1Rs and together with E100 increased the level of anandamide in specific regions of the brain,^25,26,30-32^ with acetaminophen (APAP) and URB59 enhancing social activities.^30,31^ MJN110, which is known to inhibit monoacylglycerol lipase (MAGL), increased the level of 2-AG in the prefrontal cortex with a significant decrease in the time spent in the open arm, a significant decrease in the number of entries, and a significant decrease in the time spent in the inner zone.^32^ URB59, JZL184, and Cannabidivarin downregulated most of the enzymatic components of the EC system, enhancing social activities, cognition, and repetitive behaviors.^27,28,31^ In turn, URB59, JZL184, and Cannabidivarin downregulated fatty acid amide hydrolase (FAAH), and JZL184 and Cannabidivarin downregulated MAGL.^27,28,31^

E100 and ST-2223, which are H3 receptor antagonists, together with curcumin, which is an allosteric modulator of α7-nAChR, canagliflozin, which is a sodium-glucose co-transporter type 2 (SGLT2) inhibitor, and duloxetine which belong to the class of serotonin and norepinephrine reuptake inhibitors (SNRIs), positively impacted various components of the cholinergic system with enhancement of social activities, anxiety, locomotion, and repetitive activities.^25,26,33-36^ Curcumin enhanced α7-nAChR in the hippocampus,^33^ while ST-2223 and canagliflozin increased the level of ACh in autistic animal models.^34,36^ The level of AChE was decreased in different autistic models by duloxetine and E100.^25,26,35^

Autistic animal models treated with APAP and JZL184 exhibited no significant difference in expression of CB1Rs in the prefrontal cortex.^28,30^ Apparently, JZL184 enhanced cognition, social activities, and repetitive behaviors, while APAP enhanced social activities only.^28,30^ There were no significant differences in 2-AG in the forebrain, cerebellum, and prefrontal cortex of autistic animal models when treated with URB59, E100, and PF3845, respectively.^25,26,31,32^ While social activities were enhanced in the treatment with URB59 and E100,^25,26,31^ decreased repetitive behaviors and anxiety activities were recorded when autistic animal models were treated with E100.^25,26^ In the VPA-autistic animal model, there was no significant difference in N-acylphosphatidylethanolamine-specific phospholipase D (NAPE-PLD) in the hippocampus and prefrontal cortex when treated with JZL184^28^ and there was also no significant difference in NAPE-PLD in the hippocampus when treated with cannabidivarin.^27^ Also in the VPA-autistic animal model, FAAH and diacylglycerol lipase alfa (DAGLα) showed no significant difference in prefrontal cortex and hippocampus, specifically, when treated with JZL184 and cannabidivarin.^27,28^ Treatment of VPA-autistic animal models with JZL184 and cannabidivarin enhanced cognition, social activities, and repetitive behaviors.^27,28^

**Figure 2:**
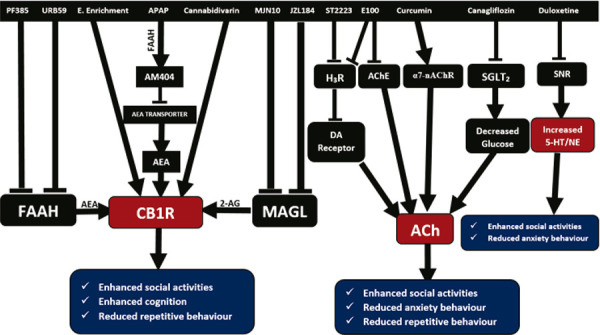
The mechanism of actions of the studied therapeutic interventions and their phenotypic effects. 5-HT/NE = 5-hydroxytryptamine/noradrenaline; ACh = acetylcholine; AChE = acetylcholinesterase; AEA = arachidonoyl ethanolamide; CB1R = type 1 cannabinoid receptor; FAA = fatty acid amide; FAAH = fatty acid amide hydrolase; MAGL = monoacylglycerol lipase; SGLT2 = sodium-glucose co-transporter type 2; SNR = serotonin and norepinephrine reuptake inhibitor; α7-nAChR = alpha 7 nicotinic acetylcholine receptor.

After APAP is broken down into p-aminophenol, it readily passes through the blood-brain barrier and is changed into AM404 by FAAH, which increases the release of anandamide.^37^ Type 1 cannabinoid receptors (CB1R) are modulated by anandamide, environmental enrichment, and cannabidivarin.^37-40^ The enzyme FAAH, which increases the release of anandamide and modifies CB1R, is effectively and irreversibly inhibited by PF-3845 and URB59.^41,42^ MJN10 and JZL184 inhibit MAGL, which in turn modifies CB1R by increasing production of 2-AG.^43-45^ ST-2223 inhibits H_3_ receptors, which in turn inhibits dopamine receptors by increasing histamine levels.^36^ E100 functions as both an AChE inhibitor and an H_3_R antagonist.^25,26^ Curcumin modulates α7-nAChR allosterically,^46,47^ canagliflozin inhibits SGLT2^48^; and duloxetine prevents SNR from being reabsorbed.^49^ The activities of ST-2223, E100, canagliflozin, and curcumin ultimately enhance ACh release, as illustrated in Figure 2.

When it comes to the findings in this systematic review, our major focus was on research that assessed how different therapeutic interventions affected components of the EC and cholinergic systems in models of autism. The behavioral activities associated with each of the three core symptoms of ASD were also highlighted, to ascertain the phenotypic impact of the therapeutic interventions (Table 1).

**Table 1:** Studies selected for the systematic review of scientific investigations targeting the cholinergic and endocannabinoid systems as a therapeutic interventions for core autism associated phenotypes

S/N	Title and authors	Therapeutic intervention	ASD model	Age/stage of the animal treatment	Brain regions	Signaling system	Therapeutic effect on ASD model signaling system compared to untreated ASD model	Therapeutic effect on ASD model behaviors compared to untreated ASD model
1	Acetaminophen differentially enhances social behavior and cortical cannabinoid levels in inbred mice Gould et al.^30^	Acetaminophen (100 mg/kg)	BTBR	Adult	PFC	CB1R	Non-significant difference	Enhanced social interaction and non-significant difference in the number of buried marbles
						AEA	Upregulated	
								
2	Enhancement of anandamide-mediated endocannabinoid signaling corrects autism-related social impairment Wei et al.^31^	URB59 (1 mg/kg)	BTBR	Young adult	Forebrain	Anandamide	Upregulated	Enhanced social approach and non-significant difference in the time spent and the number of entries in open arm
						2-AG	Non-significant difference	
						FAAH	Downregulated	
								
3	Experimental studies indicate that ST-2223, the antagonist of histamine H3 and dopamine D2/D3 receptors, restores social deficits and neurotransmission dysregulation in mouse model of autism Eissa et al.^36^	ST-2223 (5 mg/kg)	BTBR	Adult	PFC, striatum, and hippocampus	ACh	Upregulated	Enhanced social approach
								
4	Curcumin potentiates α7 nicotinic acetylcholine receptors and alleviates autistic-like social deficits and brain oxidative stress status in mice Jayaprakash et al.^33^	Curcumin (1 µM)	BTBR	Adult	CA1 region of the hippocampus	α7-nACh receptors	Potentiated α7-nACh receptors	Enhanced sociability and social preference index
								
5	Duloxetine ameliorates valproic acid-induced hyperactivity, anxiety-like behavior, and social interaction deficits in zebrafish Joseph et al.^35^	Duloxetine (4.5-6 dpf)	VPA- zebrafish	Juvenile- adult	Whole brain	AChE	Downregulated	Enhancement of social activity and reduced anxiety behavior
								
6	Canagliflozin alleviates valproic acid-induced autism in rat pups: Role of PTEN/PDK/PPAR-γ signaling pathways Elgamal et al.^34^	Canagliflozin (10 mg/kg)	VPA- Sprague-Dawley	Infant	Cerebrum, and cerebellum	ACh	Upregulated	Enhancement of social interaction and reduced anxiety behavior
								
7	The dual-active histamine H3 receptor antagonist and acetylcholine esterase inhibitor E100 alleviates autistic-like behaviors and oxidative stress in valproic acid induced autism in mice Eissa et al.^25^	E100 (10 mg/kg)	VPA- C57BL/6	Juvenile	Cerebellum	AChE	Downregulation	Enhancement of social interaction and reduced number of buried marbles and anxiety behavior
								
8	Simultaneous blockade of histamine H3 receptors and inhibition of acetylcholine esterase alleviate autistic-like behaviors in BTBR T+ tf/J mouse model of autism Eissa et al.^26^	E100 (5 mg/kg)	BTBR	Adult	Cerebellum	AChE	Downregulation	Enhanced social interaction, reduced number of buried marbles, reduced percentage escalation of shredded nestlet, and reduced anxiety behavior
						Anandamide	Upregulation	
						2-AG	Non-significant difference	
								*Continued on next page*
9	Cannabidivarin treatment ameliorates autism-like behaviors and restores hippocampal endocannabinoid system and glia alterations induced by prenatal valproic acid exposure in rats Zamberletti et al.^27^	Cannabidivarin (20 mg/kg)	VPA- Sprague Dawley rats	Juvenile- adult	Hippocampus	CB1R	Downregulated	Enhanced sociability and social preference. Enhanced short-term recognition memory and decreased grooming time.
						CB2R	Upregulated	
						FAAH	Downregulated	
						MAGL	Downregulated	
						NAPE-PLD	Non-significant difference	
						DAGLα	Non-significant difference	
								
10	Effects of environmental enrichment and sexual dimorphism on the expression of cerebellar receptors in C57BL/6 and BTBR + Itpr3tf/J mice Monje-Reyna et al.^29^	Environmental enrichment (1 h/day for 20 days)	BTBR	Adult	Cerebellum	CB1R	Downregulated	
								
11	Increasing endocannabinoid tone alters anxiety-like and stress coping behaviour in female rats prenatally exposed to valproic acid Thornton et al.^32^	PF3845 (10 mg/kg)	VPA-Sprague–Dawley rats	Juvenile	PFC	Anandamide	Upregulated	Non-significant difference in the time spent in the open arm and non-significant difference in the number of entries and the time spent in inner zone of open field test
						2-AG	Non-significant difference	
		MJN110 (5 mg/kg)	VPA-Sprague-Dawley rats	Juvenile	PFC	Anandamide	Non-significant difference	Significant decrease in the time spent in the open arm and significant decrease in the number of entries and the time spent in inner zone
						2-AG	Upregulated	
								
12	Alterations of the endocannabinoid system and its therapeutic potential in autism spectrum disorder Zou et al.^28^	JZL184 (10mg/kg)	VPA-Wistar rats	Juvenile	Hippocampus	CB1R	Downregulated	Decreased escape latency, and time of platform crosses in Morris water maze test. increased sociability and preferential index in social interaction. Decreased number of buried marbles in Marble burying test. Decreased grooming time in Self-grooming test.
						CB2R	Downregulated	
						NAPE-PLD	No significant difference	
						FAAH	Downregulated	
						DAGL	Downregulated	
						MAGL	Upregulated	
					PFC	CB1R	No significant difference	
						CB2R	Downregulated	
						NAPE-PLD	No significant difference	
						FAAH	No significant difference	
						DAGL	Downregulated	
						MAGL	Downregulated	

2-AG = 2-arachidonoylglycerol; ACh = acetylcholine; AChE = acetylcholinesterase; AEA = arachidonoyl ethanolamide; CB1R = type 1 cannabinoid receptor; CB2R = type 2 cannabinoid receptor; DAGLα = diacylglycerol lipase alfa; FAAH = fatty acid amide hydrolase; MAGL = monoacylglycerol lipase; NAPE-PLD = N-acylphosphatidylethanolamine-specific phospholipase D; PFC = prefrontal cortex; S/N = serial number; α7-nACh = α7 nicotinic acetylcholine receptor.

## Discussion

The etiology of autism is complex and involves a variety of factors, such as genetic, epigenetic, environmental, and immunological contributors, and has a heterogeneous nature.^1,4^ Abnormal changes in molecular signaling pathways, neuronal synapses, the immune environment, and functional brain connections are the ultimate manifestations of autism.^50^ An enormous regulatory network in the body maintains homeostasis and physiology through neuronal signals originating from the EC and cholinergic systems.^5-7^ ASD has been linked to dysregulated cholinergic and EC systems. In addition to their significant roles in various events in brain development, the cholinergic and EC system are also vital to a number of physiological processes and neuroadaptive responses, such as movement control, learning and memory, nociception, reward, and endocrine function.^8,9^

### Therapeutic regulation of the EC system

Recent evidence from research on humans and animals refers to the EC system’s role in the etiology of ASD. Patients with ASD have been found to have reduced EC levels in their bloodstream as well as altered EC receptors and enzymes.^5,51,52^ Human evidence showing changes in many EC system components in the brains of hereditary and environmental models of autism is supported by animal studies.^53-56^ It has been observed that pharmacological manipulation of EC signaling can improve certain animal phenotypes associated with ASD.^31,57-59^ This suggests that targeting the EC system may be advantageous in mitigating the symptoms of ASD. In line with literature data, we found that cannabidivarin, environmental enrichment, and JZL184 reversed the excessively upregulated CB1Rs in autistic animal models, with a positive impact on autistic behaviors.^27-29^ Acetaminophen, URB59, E100, PF3845, and MJN110 increased EC levels (anandamide) and ameliorated the associated autistic behaviors, except PF3845, which was associated with no significant differences in the assessed behavioral activities.^25,26,30-32^ Cannabidivarin, JZL184, and URB59 decreased the level of hydrolytic enzyme (FAAH), which is the enzyme responsible for hydrolysis of anandamide, while Cannabidivarin and JZL184 downregulated MAGL, which is a serine hydrolase that plays a crucial role in catalyzing hydrolysis of monoglyceride 2-AG into glycerol and fatty acids.^27,28,31^ Downregulation of EC system hydrolytic enzymes by cannabidivarin, JZL184, and URB59 enhanced cognition, social activities, and repetitive behaviors.^27,28,31^ The reversal of the control level of the components of the EC system reported in the reviewed literature indicates that the EC system is a strong therapeutic target for ameliorating core ASD phenotypes in clinical trials.

The results of this review indicate that pharmacological modulators of the EC system may offer therapeutic potential in ASD. The results of downregulating CB1Rs, increased degradation of EC hydrolytic enzymes, and compensatory upregulation of EC signaling molecules corroborated the reversal of ASD-associated phenotypes to the control level. The behavioral findings related to the EC system comprised reduced repetitive and stereotypical behaviors in the marble burying and self-grooming tests, reduced hyperactivity in the open field test, increased sociability and social preference in the three-chamber test, enhanced short-term recognition memory in the novel object recognition test, and improved cognitive functioning in the Morris water maze test. This review of research papers that assessed EC components is important to encourage identification of potential targets for improved therapeutic treatments in ASD.

### Therapeutic regulation of the cholinergic system

Clinical investigations have indicated that abnormalities in brain cholinergic neurotransmission may be a major factor in the behavioral aspects associated with ASD.^60^ As a result, this review focused on how novel multiple-active test substances (curcumin, ST-2223, canagliflozin, duloxetine, and E100) modulate the cholinergic system’s brain components in ASD behavioral symptoms that are observed in both genetic and environmental models of autism.

In the animal model of ASD, a reduction in ACh leads to significant changes in grooming and rearing patterns of behavior and duration,^61-63^ a rise in repetitive-stereotyped movements over time,^61,64^ social deficits, and an increase in anxiety-like behaviors. Downregulation of ACh, believed to be a neurotransmitter involved in neuronal development in the brain,^65^ has been linked to behavioral alterations in autistic patients.^66^ In both human and animal ASD research, there was an increase in expression of the AChE protein.^24,67^ Kim et al.^61^ reported increased AChE expression in cultures treated with VPA. Research by Friedman et al.^67^ showed that there were changes in the level of choline-containing compounds in many brain regions of ASD patients.

In line with the papers reviewed in this study, curcumin potentiates α7-nAChRs and along with ST-2223 and canagliflozin increases the level of ACh, which ultimately alters the ASD-associated phenotypes by enhancing social activities.^33,34,36^ Specifically in the VPA-autistic Sprague- Drawley rat model treated with canagliflozin, reduced grooming and rearing, reduced time spent in the close arm, and increased time spent in the open arm were observed in the elevated plus maze test, while reduced locomotion and grooming and increased time spent in the central area were recorded in the open field test.^34^ AChE, which is an enzyme that catalyzes the breakdown of ACh, was downregulated in various autistic models after treatment with duloxetine and E100.^25,26,35^ Tests were conducted for behaviors associated with the ASD phenotype in autistic animal models treated with E100, demonstrating enhanced social activities, reduced number of buried marbles, increased time spent and number of entries in the open arm, and decreased percentage escalation of shredded nestlets.^25,26^ Several lines of evidence suggest that EC and nicotinic cholinergic systems are implicated in the regulation of different physiological processes,^68^ including cognition, social activities, anxiety, and repetitive behaviors. The existence of crosstalk between these two systems is substantiated by the overlapping distribution of cannabinoid and nAChRs in many brain structures.^68^

The primary phenotypes linked to ASD are shown by the dysregulated components of the cholinergic and EC system.^5-7^ The ability of CB1Rs to inhibit release of ACh, which is mediated by both AChRs, causes synaptic impairments in autism due to the abnormally excessive expression of CB1Rs in several brain regions. The way these systems interact lends credence to the theory that one of the mechanisms regulating synaptic activities in a number of neuropsychiatric disorders is the control of cholinergic activity through activation of CB1R.^69^ The brain’s excitatory-inhibitory balance is influenced by cholinergic signaling. Long-term potentiation (LTP), which promotes a depolarization state, is induced by nAChRs that are postsynaptically or presynaptically situated and can increase intracellular Ca2+ release to affect synaptic plasticity.^70^ It has been reported that autistic people have altered levels of nAChRs in several different brain areas.^71^ Moreover, the cerebellum, parietal, and frontal cerebral cortex showed reduced expression levels of α4β2 nAChRs among individuals with ASD.^72-74^ It was shown, however, that the granule cell layer of the cerebellum had elevated α7nAChR subunit expression, but Purkinje cells and the molecular cell layer did not show the same effect. Research by Ray et al.,^75^ however, found that the paraventricular nucleus (PV) and nucleus reuniens (Re) had decreased neuronal α7- and β2-nAChR IR-y, and that the PV had lost its α7 neuropil IR-y. The EC system maintains major significance among the neuromodulatory systems that regulate cholinergic neurotransmission. Alterations in EC signaling have been reported in postmortem human samples from autistic patients and in animal models of cognitive impairment and cholinergic lesion models.^76^ Numerous lines of evidence indicate that the neuropathological basis of psychiatric disorders as well as the regulation of other physiological processes, including reward, are associated with the EC and nicotinic cholinergic systems.^77^ The overlapping distribution of nicotinic ACh and cannabinoid receptors in many brain regions suggests an interaction between these two systems.^77^ As such, the nicotinic cholinergic and EC systems constitute a viable pharmacological target for development of effective therapeutic interventions to treat the neuropsychiatric phenotypes linked to autism. This review outlined the impact of therapeutic modulators targeting these systems for ameliorating the core symptoms of ASD and directing the development of therapeutic interventions with potential for crosstalk between the cholinergic and EC systems.

## Conclusion

Alteration of the brain components of the cholinergic and EC system is significant in ASD-related behavior, with the results of this review indicating that pharmacological modulators of the cholinergic and EC systems may offer therapeutic potential in ASD. Pre-clinical trials of the combinations of some of these therapeutic interventions are warranted to assess their effectiveness and safety.
